# High Altitude Pregnancies and Vascular Dysfunction: Observations From Latin American Studies

**DOI:** 10.3389/fphys.2021.786038

**Published:** 2021-12-07

**Authors:** Alejandro Gonzalez-Candia, Emilio A. Herrera

**Affiliations:** ^1^Laboratorio de Función y Reactividad Vascular, Programa de Fisiopatología, Instituto de Ciencias Biomédicas, Facultad de Medicina, Universidad de Chile, Santiago, Chile; ^2^Instituto de Ciencias de la Salud, Universidad de O'Higgins, Rancagua, Chile; ^3^International Center for Andean Studies (INCAS), Universidad de Chile, Santiago, Chile

**Keywords:** gestation, chronic hypoxia, hypobaria, endothelial dysfunction, cardiovascular, placenta, fetal programming

## Abstract

An estimated human population of 170 million inhabit at high-altitude (HA, above 2,500 m). The potential pathological effects of HA hypobaric hypoxia during gestation have been the focus of several researchers around the world. The studies based on the Himalayan and Central/South American mountains are particularly interesting as these areas account for nearly 70% of the HA world population. At present, studies in human and animal models revealed important alterations in fetal development and growth at HA. Moreover, vascular responses to chronic hypobaria in the pregnant mother and her fetus may induce marked cardiovascular impairments during pregnancy or in the neonatal period. In addition, recent studies have shown potential long-lasting postnatal effects that may increase cardiovascular risk in individuals gestated under chronic hypobaria. Hence, the maternal and fetal adaptive responses to hypoxia, influenced by HA ancestry, are vital for a better developmental and cardiovascular outcome of the offspring. This mini-review exposes and discusses the main determinants of vascular dysfunction due to developmental hypoxia at HA, such as the Andean Mountains, at the maternal and fetal/neonatal levels. Although significant advances have been made from Latin American studies, this area still needs further investigations to reveal the mechanisms involved in vascular dysfunction, to estimate complications of pregnancy and postnatal life adequately, and most importantly, to determine potential treatments to prevent or treat the pathological effects of being developed under chronic hypobaric hypoxia.

## Introduction

An estimated human population of 170 million inhabit at high-altitude (HA, above 2,500 m) (Moore et al., 1998; Herrera et al., 2015). The pathological effects of HA hypobaric hypoxia during gestation have been the focus of several researchers worldwide, specifically in the cardiovascular and respiratory systems. The studies based on the Himalayan and Central/South American mountains are of particular interest, as these areas account for nearly 70% of the HA world population. Furthermore, cities with the higher population density at HA are located in the Andean Mountains (Moore et al., 1998). At present, studies in human and experimental animal models revealed significant impairments in fetal development and growth at HA. Moreover, vascular responses to chronic hypobaria in the pregnant mother and her fetus may induce marked cardiovascular dysfunctions during pregnancy or in the neonatal period (Herrera et al., 2015). In addition, recent studies have shown potential long-lasting postnatal effects that may increase cardiovascular risk in individuals gestated under chronic hypobaria (Ducsay et al., 2018). The maternal, placental, and fetal adaptive responses to hypoxia are vital for a better developmental and cardiovascular outcome at birth. Still, these responses are generally accompanied by an increased risk of developing chronic non-communicable diseases.

## Pregnancy at High-Altitude Hypobaria

Several environmental alterations occur at HA, such as increased radiation, decreased environmental temperature and humidity, and reduced barometric pressure. The latter is considered the most important for pregnancy development as it impacts the environmental oxygen pressure (PO_2_) and, therefore, the oxygen (O_2_) availability for the mother and fetus (Julian, 2011; West, 2017). In fact, at altitudes higher than 2,500 m above sea level, most mammals (including humans) undergo decreased O_2_ saturation and hypoxemia (Moore, 2001; Herrera et al., 2015). This chronic hypoxemia during pregnancy drives several maternal, placental, fetal, and postnatal consequences that affect oncoming health (Yzydorczyk et al., 2017; Ducsay et al., 2018; Moore, 2021).

## Maternal Effects

**Figure 1:**
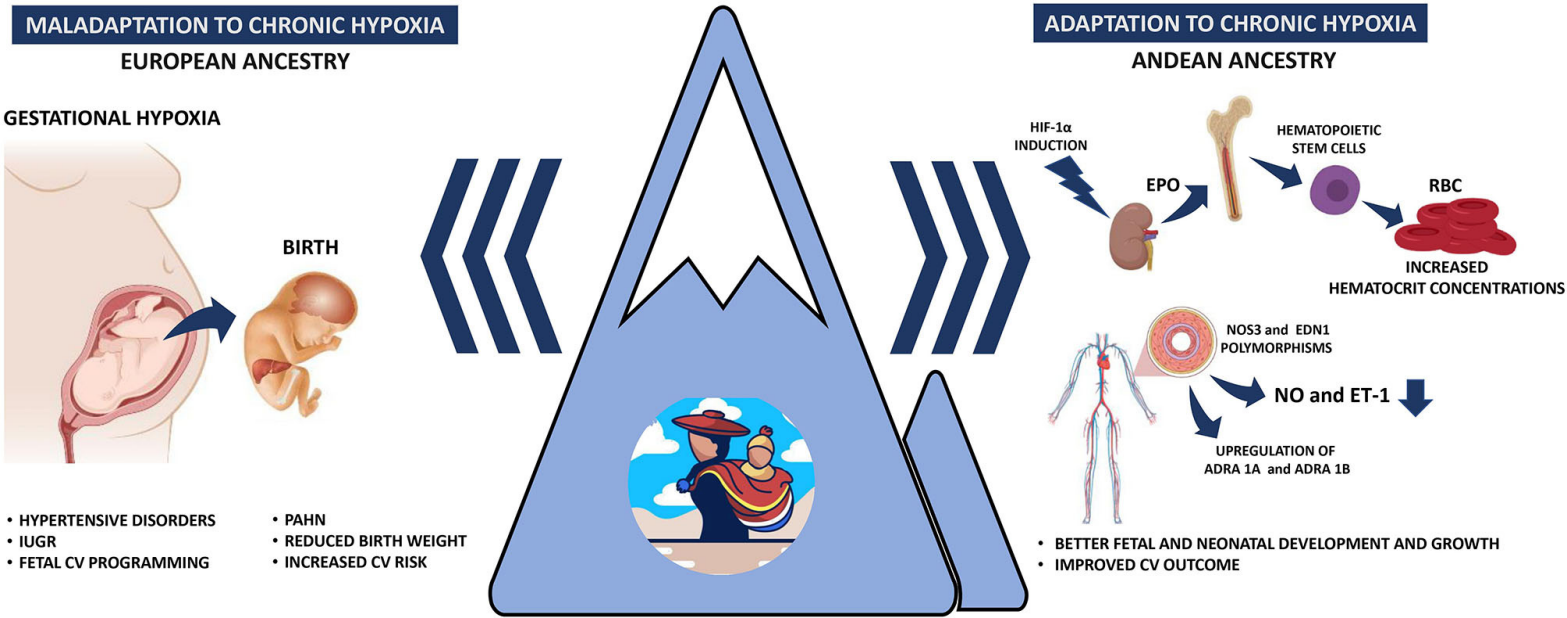
Schematic representation of the gestational responses under hypobaric hypoxia. The left panel represents the gestational and perinatal consequences of a non-adapted (European ancestry) population to hypobaric hypoxia. The right panel depicts the Andean population adaptations to increase tissue oxygenation, increase hemoglobin concentration, and differentially expression of vascular mediators. IUGR, intrauterine growth restriction; PAHN, pulmonary arterial hypertension of the neonate; HIF-1α, hypoxia-inducible factor 1α; EPO, erythropoietin; RBC, red blood cells; NO, nitric oxide; ET-1, endothelin-1.

Maternal cardiovascular responses to HA have been proposed as main drivers of the pregnancy complications at HA, such as systemic hypertension, bleeding, oligoamnios, placental insufficiency, preterm labor, and IUGR (Keyes et al., 2003; Lorca et al., 2019; Bailey et al., 2020; Figure 1). HA pregnancies have a ~30% increased frequency of hypertensive disorders when comparing similar populations (Bailey et al., 2020; Grant et al., 2021) and specifically duplicate gestational hypertension (Grant et al., 2021). However, controversies have been reported in preeclampsia (PE) prevalence in HA populations. While some studies reveal an increased frequency of PE, up to 16% (Palmer et al., 1999; Keyes et al., 2003; Bailey et al., 2020), others have shown a decreased risk of developing PE at HA (Grant et al., 2021). This discrepancy in PE prevalence may be due to ethnic differences in the studied population. For example, HA pregnancies in Andeans vs. Europeans have higher estrogen levels, possibly a protective factor for PE (Charles et al., 2014). In addition, Andean residents have increased antioxidant capacity and diminished oxidative stress during pregnancy, compared to European residents at HA (Julian et al., 2012). Also, Andeans, on average, have an increased uterine blood flow and O_2_ delivery during pregnancy (Julian et al., 2009). Conversely, an enhanced systemic vascular response to chronic hypoxia in HA newcomers may induce maternal hypertensive disorders and PE (Ahmed et al., 2017; Tejera et al., 2021; Figure 1). Based on the little evidence, it seems that hypertensive disorders during pregnancy are more common at HA, but multigenerational residents appear to be protected relative to newcomers. Still, more studies are needed to clarify this protection and the involved mechanisms.

HA impairs maternal and fetal vasodilatory capacity by several pathways. One of the most affected mechanisms is the nitric oxide(NO)-dependent arterial vasodilation because chronic hypoxia determines endothelial dysfunction and oxidative stress, both enhanced at HA (Herrera et al., 2014, 2015). However, this decreased NO function did not affect the total vasodilation capacity of myometrial arteries and showed an enhanced prostanoid vasodilator function in pregnant women (Lorca et al., 2019). The enhancement of alternative vasodilator pathways may be an important protective in maternal and fetal cardiovascular response, such as prostacyclin, endothelium-derived hyperpolarizing factor (EDHF), carbon monoxide (CO), or hydrogen sulfide (H_2_S) (Yzydorczyk et al., 2017; Gao and Galis, 2021). However, these vasodilator pathways have been widely unexplored at HA.

## Maternal Acclimatization and Adaptation

Several studies have described short-term responses or acclimatization (newcomers) and long-term responses or adaptation (evolutionary) to HA in the Andean population (Moore, 2017). Most of the cardiovascular and pulmonary adaptive mechanisms in the Andean population account for an improved O_2_ transport delivery and metabolism. Conversely, adapted Andeans are characterized by having larger lung volumes, diminished PAO_2_ to PaO_2_ gradients, less hypoxic pulmonary vasoconstrictor response, enhanced uterine artery blood flow during pregnancy, increased birth weight and cranial circumference, and improved cardiac O_2_ utilization relative to lowlanders (Bennett et al., 2008; Moore et al., 2011; Pizarro-Ortiz et al., 2014; Julian and Moore, 2019), suggesting an overall greater efficiency of O_2_ transfer and utilization.

Furthermore, Andean ancestry determines enhanced adaptations to HA and therefore, better pregnancy outcomes. For instance, Andeans have significantly higher live births to miscarriages ratios than Mestizo or European women in La Paz, Bolivia (Grant et al., 2020). Moreover, the impact of HA on fetal growth and birth weight is independent of economic status (Giussani et al., 2001). In addition, HA-maternal ancestry is a strong determinant of pregnancy outcome, but not the paternal ancestry (Soria et al., 2013; Grant et al., 2020).

Pregnant Andeans have an increased uterine artery diameter and blood flow, with enhanced uteroplacental O_2_ delivery, associated with an enhanced fetal growth compared to European women exposed to HA hypoxia (Wilson et al., 2007). Furthermore, babies with heavier birth weights and greater ponderal indices were born to Andean women with higher ventilation, respiratory frequency, and lower tidal volume during pregnancy (Vargas et al., 2007).

The HA exposure during many generations has induced a positive natural selection in several genes. For instance, egl-9 homolog 1 (*EGLN1)*, a gene that encodes for prolyl hydroxylase 2 enzyme (PHD2), key in the O_2_ sensing and the hypoxia-inducible factor 1α (HIF-1α) degradation were found at higher frequency in Quechua compared with lowland populations (Bigham et al., 2010; Brutsaert et al., 2019). In addition, Andeans have low endothelin-1 (ET-1) levels, a potent vasoconstrictor induced by HIF-1, during low or high-altitude pregnancies. However, European mothers markedly increased plasma ET-1 levels at HA (Moore et al., 2004). Moreover, several key HIF-regulatory and targeted genes are responsible for adaptation to HA in Andeans (reviewed elsewhere by Bigham, 2016). Furthermore, single nucleotide polymorphisms (SNPs) associations with birth weight have been identified near coding regions for two genes, *Protein Kinase AMP-Activated Catalytic Subunit Alpha 1* (*PRKAA1)* and *Endothelin Receptor Type A (EDNRA)*, implicated in O_2_ sensing and vascular control (Bigham et al., 2014). In addition, the authors found significant associations of these SNPs with vascular protection and HA-associated fetal growth restrictions. These adaptations have presumably been selected in the last 10,000–15,000 years of human populations living at the Andean Altiplano (Aldenderfer, 2003).

Altogether, the maternal responses to achieve a successful pregnancy at HA will, therefore, depend on the altitude-ancestry characteristics and the individual adaptive capacity (Figure 1).

## Fetal Effects

### Growth

The most frequent fetal finding at HA pregnancies is intrauterine growth restriction, with marked decreases in fetal size and weight relative to lowland gestations. Interestingly, HA ancestry is closely related to birth weight when comparing different ethnicities at similar altitudes (Moore, 2001). The decline in birth weight is markedly less in Tibetans (~88 g each 1,000 m) or Andeans (~89 g each 1,000 m) than in Europeans (~119 g each 1,000 m) and Han (~153 g each 1,000 m) (Moore et al., 2011). In addition, several other studies show that Andean ancestry raises birth weight (Giussani et al., 2001; Bennett et al., 2008; Soria et al., 2013). Therefore, multigenerational Andean residents are relatively protected from altitude-associated fetal growth reductions (Julian et al., 2007, 2011; Pizarro-Ortiz et al., 2014; Figure 1.

### Cardiovascular Effects

Development under chronic hypoxia induces marked cardiovascular effects, particularly in the placental, umbilical and pulmonary circulation, causing vascular hypertension, oxidative stress and remodeling (van Patot et al., 2012; Herrera et al., 2014). As placental and pulmonary vascular beds are susceptible to O_2_ drop, HA hypoxia induces permanent effects during pregnancy and after birth. Being born in a low-oxygen environment delays an adequate perinatal pulmonary transition, resulting in impaired respiratory reflexes and enhanced pulmonary vasoreactivity (Keyes et al., 2003; Niermeyer, 2003). Furthermore, a systemic review showed a delayed postnatal remodeling of the pulmonary artery, positively correlating the altitude to the magnitude of the pulmonary arterial pressure (Peñaloza and Arias-Stella, 2007). In addition, the same study revealed an increased thickness of the right ventricle in the neonatal and infant periods of children born and raised at HA. In contrast, a cross-sectional study performed at 4,000 m with echocardiography indicate that < 1% of the cardiac problems are attributable to HA (Huicho et al., 2005; Huicho and Niermeyer, 2006). However, most children of the studied population had at least a certain degree of high-altitude genetic ancestry. Authors conclude that the very low cardiopulmonary alterations found are due to the protective effect of several factors, including ancestry, good health and nutritional status, and low indoor pollution, among others (Huicho et al., 2005; Huicho and Niermeyer, 2006). Moreover, the cerebral circulation is also affected by a marked decrease in O_2_ saturation compensated with a higher vessel density (Gassmann et al., 2016). Still, further studies are needed to assess the real influence of chronic hypobaric hypoxia on the early postnatal cardiovascular development at high altitudes.

In summary, babies and children born and raised at HA are at particular risk for hypoxemia, pulmonary arterial hypertension, persistence of fetal vascular connections, and cardiovascular remodeling. However, more studies are needed to develop reliable diagnostic and predictive health outcomes.

## Long-Lasting Cardiovascular Effects (Programming)

The heart and pulmonary circulation of individuals gestated, born and living at HA exhibit important physiologic and anatomic characteristics to maintain O_2_ homeostasis, such as alveolar hypoxia, hypoxemia, polycythemia, increased vascular density, and metabolic reprogramming (Prabhakar and Semenza, 2012). However, when these physiological adaptations are permanent at HA, neonates and children can generate pulmonary hypertension and right ventricular hypertrophy as compensatory mechanisms to environmental hypoxia (Peñaloza and Arias-Stella, 2007). Many of the physiological responses to chronic hypoxia are mediated at the transcriptional level by the binding of HIF-1 to the hypoxia-response element (HRE) located in the target gene (Wenger et al., 2005). *EPO*, which encodes erythropoietin, is one of the best characterized hypoxia-induced genes. Erythropoietin expression induces red blood cell proliferation by inhibiting apoptosis in erythroid progenitors, increasing blood O_2_-carrying capacity (Jelkmann, 2004). However, among ethnic groups living at HA, only Andeans have increased hemoglobin (Hb) concentrations (Nanduri et al., 2017). These phenotypic differences have been elucidated through epigenetic mechanisms, which are changes in gene promoter and chromatin that regulate gene transcription by altering the accessibility of the DNA for transcription factors without changes in the coding sequence of DNA (Feinberg, 2007). Particularly, the *EPO* gene promoter methylation of the CpG island is increased and negatively correlated with gene expression in Tibetans and Ethiopians (Yin and Blanchard, 2000). The higher perinatal Hb concentration in Andeans is associated with an increased pulmonary vascular dysfunction and a greater incidence of chronic mountain sickness (CMS) during adulthood (Julian et al., 2015).

On the other hand, *EGLN1* encodes for the PHD2 enzyme, which negatively regulates the stability of HIF-1α in an oxidative stress and O_2_-dependent manner. In addition, endothelial PAS domain protein 1 (*EPAS1)*, which encodes HIF-2α, has been implicated in human evolutionary adaptation to HA (Simonson et al., 2010). Mutations or dysregulation of these genes or their products has been associated with anemia and polycythemia (Lorenzo et al., 2014). In addition, variation at the *EGLN1* locus is associated with protection against polycythemia and low pulmonary vasoconstriction response in highlanders (Peng et al., 2017). One of the proposed mechanisms is the downregulation of the angiotensin-converting enzyme (ACE) and vascular endothelial growth factor C (VEGFC) in the heart and the lungs (Peng et al., 2017). ACE generates the vasoconstricting peptide angiotensin II in the lung and vascular endothelium and inactivates the vasodilating peptide bradykinin, among other mechanisms (Chappell, 2016). Therefore, by inhibiting the bioavailability of angiotensin II in the vascular endothelium, the vasoactive balance shift toward vasodilation, decreasing pulmonary arterial pressure as observed in Tibetans. However, this adaptation is not found in Andeans (Azad et al., 2017). *VEGF* is a target gene of *EPAS1* and plays an essential role in angiogenesis; the upregulation of VEGF is involved in vascular remodeling associated with the development of pulmonary hypertension induced by chronic hypoxia (Liu et al., 2018). Again, Tibetan is the only population known to negatively regulate the *VEGF* gene compared to the Andeans and Ethiopians (Peng et al., 2017). Effectively, Tibetans have occupied the HA plateau for at least 20,000 years, having the most prolonged exposure to HA and, therefore, a stronger evolutionary adaptive capacity (Aldenderfer, 2003).

The vascular tone is regulated by a balance between the effects of vasodilators/antiproliferative and vasoconstrictors/mitogenic agents (Said, 2006). However, the hypobaric hypoxia increased vascular tone as a result of decreased levels of second messengers such as cyclic guanine monophosphate (cGMP) and cyclic adenine monophosphate (cAMP), inactivation of K+ channels, and increased levels of endogenous vasoconstrictors (e.g., ET-1) and reactive oxygen species (ROS) (Moudgil et al., 2005). The major inducer of cGMP-mediated vasodilation is nitric oxide (NO) at the vascular level; however, only the Tibetan population has an increase in the concentration of NO with > 10-fold-higher circulating concentrations of bioactive NO products (Erzurum et al., 2007). One possible hypothesis is that native American people possess polymorphisms for the *NOS3* gene that codes for eNOS. The 894T variant encodes for aspartic acid instead of glutamic acid, which renders the enzyme inactive, depleting NO levels (Mishra et al., 2015). Likewise, the 4a variant, also termed short intronic repeats RNA, seems responsible for decreasing *NOS3* expression through miRNA-mediated inhibition (Zhang et al., 2008). On the other hand, Tibetan and Andean populations have lower ET-1 levels than lowland populations due to the prevalence of ET-1 longer repeats of (CT)n-(CA)n in the 5=-untranslated region (UTR) microsatellite and G allele of 2288G/T (rs2070699) intronic polymorphism (Mishra et al., 2015).

In addition to the covalent modifications of the chromatin or the promoter regions of a gene, the regulation of gene expression through miRNA is less studied in the highlander population. miRNAs such as miR-210,−26, and−181 regulate HIF to modify endothelial cell response to hypoxia, contributing to cell proliferation, angiogenesis, vasoactive response, metabolism programming, and cellular survivor (Suarez et al., 2007; Crosby et al., 2009; Bertero et al., 2017). On the other hand, miR-21 have been implicated as regulators in the etiology of pulmonary hypertension, downregulating BMP receptor type 2 signaling (Parikh et al., 2012) or downregulating the expression of eNOS, decreasing the bioavailability of NO (Peñaloza et al., 2020). Although it was initially thought that epigenetics modifications were irreversible, now we know that several of the involved mechanisms may be modified by environmental conditions, such as cellular metabolism, oxygen availability, and redox status during lifespan (Lamadema et al., 2019). Unfortunately, we cannot predict if the physiological adaptations and functional CV effects are permanent or they can be reverted. However, chronic cardiovascular alterations such as cardiopulmonary remodeling associated with vascular SMC hypertrophy and wall fibrosis are, until now, irreversible and progressive under chronic hypoxia (Herrera et al., 2015; Sydykov et al., 2021). Investigating the epigenetic mechanisms and their functional effects in HA populations will help explain their roles in the adaptive strategies these populations possess to cope with hypobaric hypoxia (Figure 1).

## Treatments and Future Perspectives

Descend to lower altitudes is the most effective and only treatment for HA disorders in pregnant women. However, several therapies are available for adults (men and non-pregnant women), with acetazolamide and dexamethasone (Dex) being the two best-tested drugs used as preventive medication during HA exposure (Joyce et al., 2018; Sydykov et al., 2021). Acetazolamide, a potent carbonic anhydrase inhibitor, induces metabolic acidosis leading to the activation of peripheral chemoreceptors and increased ventilatory drive during exposure to low oxygen pressure (Toussaint et al., 2021). On the other hand, Dex blocks the arachidonic acid pathway, decreasing inflammatory mediators like systemic prostaglandins (O'Hara et al., 2014). Furthermore, Dex reduces lung vascular permeability and transmural microvascular pressure by increasing surfactant into the alveolar tissue. All these effects result in decreases in pulmonary arterial pressure (Bliss et al., 2019). However, the administration of acetazolamide and Dex during pregnancy is contraindicated, and FDA only recommended its use if the potential maternal benefit outweighs the potential fetal risk.

Conversely, if complications occur during the perinatal period, therapy is based on O_2_ treatment and inhaled NO. The vasodilatory function of NO is mediated by cGMP (Li et al., 2018), but NO may fail due to increased enzyme phosphodiesterase type 5 (PDE5) that hydrolyzes cGMP. Sildenafil and Tadalafil, both PDE5 inhibitors, provide an acute pulmonary vasodilatory effect and improve gas exchange, which might prevent hypoxic pulmonary vasoconstriction (Xia et al., 2014; Sydykov et al., 2021). Other novel therapies have been proposed to be tested in HA pulmonary hypertension, such as Fasudil, a rho kinase inhibitor. Rho-kinase is an enzyme that plays an important role in mediating vasoconstriction and vascular remodeling in the pulmonary bed stimulated by hypobaric hypoxia (Kojonazarov et al., 2012). In addition, Riociguat, an sGC stimulator, may also be useful for pulmonary arterial hypertension at HA (Beghetti et al., 2019). As with other approved therapies for pulmonary arterial hypertension, Riociguat has antifibrotic, antiproliferative, and anti-inflammatory effects, in addition to the vasodilatory properties (Beghetti et al., 2019; Klinger et al., 2020). Still, as there are no adequate and well-controlled studies to prove their effectiveness and safety in women, these drugs are currently contraindicated during pregnancy.

As chronic hypoxia induces oxidative stress, antioxidants such as vitamins C and E, coenzyme Q10 and melatonin, have been proposed for adults, pregnant women, and neonates to improve CV function at HA (Herrera et al., 2015; Biuomy et al., 2020; Giussani, 2021), These novel therapeutic strategies have shown effectiveness in animal models, but there is still a lack of clinical studies in humans.

Still, further experiments are needed to test the relevance of these treatments at HA, particularly during pregnancy.

In summary, studies at HA have enlightened our understanding of the mechanisms involved in human adaptation to chronic hypoxia. Recognizing the importance of early-life events for lifelong health promises to improve our understanding of health determinants later in life when living at HA.

## Conclusion

Studies in Andean populations have revealed several detrimental consequences of living at HA. In addition, these populations have been able to develop strategies to adapt to the HA thin air. However, the physiological processes that contribute to fetal growth restriction and further health or disease programming at altitude are still poorly understood, and thus our intervention capacity remains limited. The complete comprehension of human adaptation to HA during pregnancy includes maternal, placental, fetal determinants and their interactions. In addition, understanding HA responses may be extrapolated to sea-level pathologies affected by hypoxia such as placental insufficiency, PE, and persistent pulmonary hypertension of the neonate. Moreover, future studies aiming to understand and integrate these responses will allow us to develop public policies and clinical strategies aiding for a healthy and long-lasting life at high-altitude.

## Author Contributions

AG-C and EH drafted, edited, and approved the submitted version of the manuscript.

## Funding

This work was funded by Fondecyt de Inicio grant no. 11200798 and Fondecyt Regular grant no. 1201283.

## Conflict of Interest

The authors declare that the research was conducted in the absence of any commercial or financial relationships that could be construed as a potential conflict of interest.

## Publisher's Note

All claims expressed in this article are solely those of the authors and do not necessarily represent those of their affiliated organizations, or those of the publisher, the editors and the reviewers. Any product that may be evaluated in this article, or claim that may be made by its manufacturer, is not guaranteed or endorsed by the publisher.
